# Genome‐wide DNA methylation profile analysis in thoracic ossification of the ligamentum flavum

**DOI:** 10.1111/jcmm.15509

**Published:** 2020-06-24

**Authors:** Tianqi Fan, Xiangyu Meng, Chuiguo Sun, Xiaoxi Yang, Guanghui Chen, Weishi Li, Zhongqiang Chen

**Affiliations:** ^1^ Department of Orthopaedics Peking University Third Hospital Beijing China

**Keywords:** 850K array, DNA methylation, osteogenesis, pyrosequencing, thoracic ossification of the ligamentum flavum

## Abstract

Thoracic ossification of the ligamentum flavum (TOLF) causes serious spinal canal stenosis. The underlying aetiology may relate to genetic and inflammatory factors. DNA methylation plays a critical role in osteogenesis and inflammation, whereas there is no genome‐wide DNA methylation analysis about TOLF. The two subtypes of TOLF (single‐level and multiple‐level) have distinct clinical features. Using micro‐computed tomography (micro‐CT), we showed the ossification arose from the joint between two vertebrae at one/both sides of ligament flavum. With Illumina Infinium Human Methylation 850 BeadChip arrays, genome‐wide DNA methylation profile was measured in ligament flavum of eight healthy and eight TOLF samples. Only 65 of the differentially methylated cytosine‐phosphate‐guanine dinucleotides were found in both subtype groups. Principal component analysis and heat map analysis showed a different methylation pattern in TOLF samples, and methylation patterns of two subtypes are also distinct. The Gene Ontology enrichment analysis was significantly enriched in differentiation and inflammation. Pyrosequencing analysis and quantitative real‐time polymerase chain reaction were performed to validate the arrays results and expression levels, to test six differentially methylated genes (*SLC7A11*, *HOXA10*, *HOXA11AS*, *TNIK*, homeobox transcript antisense RNA, *IFITM1*), using another independent samples (*P* < 0.05). Our findings first demonstrated an altered Genome‐wide DNA methylation profile in TOLF, and implied distinct methylated features in two subtypes.

## 1 INTRODUCTION

Thoracic ossification of the ligamentum flavum (TOLF) is a type of pathological heterotopic ossification that can cause severe thoracic myelopathy in Asian populations. Epidemiological studies have demonstrated that TOLF frequently occurs in the thoracic spine, and over 70% of patients suffering from thoracic spinal stenosis have been diagnosed with TOLF.^1^ Due to the insidious progressive nature of the ossification and lack of conservative treatment strategies, TOLF generally requires aggressive surgery.^1^, ^2^, ^3^, ^4^ The pathological process of TOLF involves the differentiation of fibroblasts into osteoblasts, which is a highly regulated development process and can be described histologically based on endochondral ossification.^5^ Several investigations have suggested that the potential factors associated with TOLF mainly include mechanical effects,^6^, ^7^ inflammatory factors^8^, ^9^ and genetic factors.^10^, ^11^ Recently, studies have shown that inflammation and endochondral ossification play an important role in TOLF.^5^, ^12^ Several evidences have proven the involvement and possible mechanism of tumour necrosis factor α in TOLF, suggesting the contribution of inflammation in TOLF.^8^, ^9^ However, the mechanisms underlying the development of TOLF have not yet been clarified.

TOLF is usually classified into single‐level and multiple‑level TOLF based on the lesion distribution in the magnetic resonance imaging (MRI), with distinct clinical features. Single‐level TOLF occurs mainly in the lower thoracic spine (T10‐T12),^4^ while multiple‑level TOLF presents with a wide and serious ossification in immobile as well as the mobile segments that differs from single‑level lesions in terms of disease progression and clinical outcomes.^13^, ^14^, ^15^ One of our previous studies has shown that the differences between the osteogenic differentiation potency in single‐ and multiple‐level TOLF may be related to differences in the disease pathogenesis and genetic backgrounds of patients.^7^ However, the pathological differences between single‐ and multiple‐level TOLF remain unknown. Therefore, it is important to figure out whether single‐ and multiple‐level TOLF should be analysed separately, which would make TOLF research more reasonable and conducive in the future.

DNA methylation, in which the cytosine residue in cytosine‐phosphate‐guanine dinucleotides (CpGs) acquires a methyl group, is an important epigenetic mechanism. Several multiple rheumatic and autoimmune diseases, such as ankylosing spondylitis (AS), osteoporosis and osteoarthritis, show abnormal DNA methylation.^16^, ^17^, ^18^ However, few studies have reported abnormally methylated loci in patients with TOLF. A Japanese research found that decreased DNA methylation in the promoter regions of the *WNT5A* and *GDNF* genes may indirectly promote the osteogenicity of mesenchymal stem cells (MSCs) from patients with ossified spinal ligaments.^19^ And genome‐wide DNA methylation profiling of TOLF and healthy ligamentum flavum has not been performed. Meanwhile, the differences between single‐ and multiple‐TOLF with regards to DNA methylation are yet to be elucidated.

In this study, we first evaluated the morphological characteristics of TOLF by micro‐computed tomography (micro‐CT) to investigate the ossification patterns in single‐ and multiple‑level TOLF. Then we performed a comparative analysis of the genome‐wide DNA methylation profiles of ligamentum flavum samples from patients with TOLF and healthy controls. Genome‐wide DNA methylation was measured to assess the communalities and discrepancies of the ligamentum flavum methylome in patients with TOLF (single‐ and multiple‑level TOLF) and healthy controls. Furthermore, this is the first study investigating the genome‐wide epigenetic landscapes of the ligamentum flavum from patients with TOLF, and reveals the potential epigenetic differences between the two types of TOLF.

## 2 MATERIALS AND METHODS

### 2.1 Patient specimens

This study was approved by the Ethics Committee for Human Subjects of the Peking University Third Hospital in accordance with the Declaration of Helsinki (PUTH‐REC‐SOP‐06‐3.0‐A27, #2014003). Written consent was obtained from all subjects. The diagnosis of TOLF was made on the basis of clinical symptoms and radiological examination. We previously established a TOLF classification scheme based on the lesion distribution observed in the MRI analysis^7^ as follows: single‐level TOLF (ossification in two or fewer adjacent levels) and multiple‐level TOLF (continuous or intermittent ossification in three or more levels). We investigated patients with TOLF who underwent decompressive laminectomy between January 2018 and January 2019, and a total of 32 patients were enrolled in this study.

For methylation chip, there were four patients each with single‐ or multiple‐level TOLF, and eight non‑TOLF specimens were obtained from patients with other spine diseases, such as trauma or disc herniation, who had no ossification in any spinal ligaments. To elucidate the distinct methylation patterns appearing in the ligament flavum samples, we divided the patients into four groups: multiple‐healthy group (four patients with multiple‐level TOLF: four healthy controls), single‐healthy group (four patients with single‐level TOLF: four healthy controls), multiple + single/healthy group (all eight patients with TOLF: all eight healthy controls) and multiple‐single group (four patients with single‐level TOLF: four patients with multiple‐level TOLF). As the patients' age and gender may affect DNA methylation features, all the selected patients were around 60 years old (60.19 ± 1.53) in each group, and the number of men and women was the same.

And other independent samples of eight patients and eight healthy subjects were collected for validating in Pyrosequencing analysis. Patients with cancer, posterior longitudinal ligament ossification, diffuse idiopathic skeletal hyperostosis, AS, rheumatoid arthritis and other systemic autoimmune diseases were excluded from this study. Participant details are listed in Table 1. All ligament samples were aseptically obtained from patients during surgery, and were separated from non‑ossified sites to avoid any possible contamination of osteogenic cells.

**TABLE 1:** Basic characteristics of the study subjects

Clinical characteristics of TOLF subjects			Clinical characteristics of healthy controls		
Patient/group	Gender	Age	Patient/group	Gender	Age
*Methylation chip*					
Multiple‐healthy group					
m1	Male	59	h1	Male	60
m2	Male	62	h2	Male	62
m3	Female	60	h3	Female	59
m4	Female	61	h4	Female	61
Single‐healthy group					
s1	Male	62	h5	Male	61
s2	Male	60	h6	Male	56
s3	Female	59	h7	Female	62
s4	Female	58	h8	Female	59
*Pyrosequencing/qRT‐PCR analysis*					
Multiple‐healthy group					
m5	Male	61	h9	Male	62
m6	Male	59	h10	Male	58
m7	Female	60	h11	Female	61
m8	Female	62	h12	Female	60
Single‐healthy group					
s5	Male	61	h13	Male	62
s6	Male	58	h14	Male	58
s7	Female	60	h15	Female	60
s8	Female	62	h16	Female	61

Abbreviations: qRT‐PCR, quantitative real‐time polymerase chain reaction; TOLF, thoracic ossification of the ligamentum flavum.

### 2.2 Micro‐CT evaluation

The lamina was resected integrally by piezosurgery to ensure the collection of the ossified ligamentum flavum samples without damage. All the lamina specimens were scanned by micro‐CT (Inveon, Siemens Medical Solutions, USA), with a scanning space resolution of 18 μm (80 kVp, 80 μA, 900 ms exposure). Inveon Research Workplace (version 3.0, Inveon) was utilized to manually draw the region of interest (ROI) around the sites of the ossified apophysis and calcification in the ligamentum flavum in each image. These polygonal contours were then used to generate a three‐dimensional ROI for subsequent analysis and the calculation of the morphological parameters.

### 2.3 DNA isolation

DNA was isolated using the QIAamp DNA Mini kit (250) (Qiagen, Hilden, North Rhine‐Westphalia, Germany), according to the manufacturer's protocol.

### 2.4 Methylation arrays

DNA was bisulphite‐converted using the Zymo EZ DNA Methylation Kit (Zymo Research, Irvine, CA, USA) according to the manufacturer's standard protocol. Bisulphite‐converted DNA was analysed on an Illumina Infinium Methylation EPIC 850K BeadChip (Illumina, San Diego, CA). Microarray data were extracted, and the DNA methylation level was calculated using GenomeStudio Methylation Module v1.8 software (version 2011.1) with default parameters. Data were normalized by subtracting the background value, which was determined by averaging the signals of built‐in negative control bead types. The normalized data were then used to calculate the DNA methylation levels, which were displayed as β‐values ranging from 0 to 1, corresponding to unmethylated and methylated sites, respectively.

### 2.5 Pyrosequencing analysis

The DNA extraction protocol was the same as the 850 K array screening subjects. DNA was bisulphite‐converted using the EpiTect Bisulfite Kit (Qiagen) according to the manufacturer's standard protocol. Bisulphite‐converted DNA was amplified by polymerase chain reaction (PCR) using the PyroMark PCR Kit (Qiagen) in a total reaction volume of 25 μL, which contained sequencing primer (0.3 mmol/L) and 50 ng bisulphite‐converted DNA, with PCR primers listed in Table S1. After purification, 20 μL PCR product was pyrosequenced using the PyroMark Gold Q96 Kit (Qiagen) and PyroMark Gold Q96 pyrosequencer (Qiagen) according to the manufacturer's instructions. Data were collected and analysed using the PyroMark Q96 software (version 2.5.8, Qiagen).

### 2.6 Cell culture and quantitative real‐time PCR

Cell culture was conducted according to protocol as we described previously.^5^, ^9^ Total RNA was extracted with TRIzol reagent (Invitrogen Corporation, CA, USA), and cDNAs were synthesized with a SuperScript III First‐Strand Synthesis System for Reverse transcription (Invitrogen Corporation). Quantitative real‐time PCR (qRT‐PCR) for the mRNA level was carried out as described previously,^5^, ^9^ with primer pairs listed in Table S2. SYBR Green I was used for qRT‐PCR according to the manufacturer's instructions (TaKaRa, Tokyo, Japan) with the Bio‐Rad iQ5 system (Bio‐Rad, California, USA). The relative gene expression levels were calculated using the 2^‐ΔΔCt^ method. All experiments were performed in triplicate.

### 2.7 Statistical analysis

Data were presented as the mean ± SEM. Statistical differences in continuous variables between two groups were compared by the Student's *t* test. *P* < 0.05 was considered to be statistically significant. The significantly different methylation loci were defined by a threshold of |Delta_ Beta value| > 0.17 and |DiffScore| > 13 (the calculation of Delta_ Beta value is the result of the difference of Avg_Beta between control group and case group, which is the difference of methylation at each locus between experimental group and control group). A diff score for a probe is computed as: DiffScore = 10*sgn (βcond − βref)*log10(*P*), |DiffScore| > 13, equivalent to *P* < 0.05.) Gene Ontology (GO, http://www.geneontology.org) and the Kyoto Encyclopedia of Genes and Genomes (KEGG, http://www.kegg.jp/) pathway enrichment analysis were performed using the scripts in Python to clarify the function and biological pathways of differentially expressed methylation loci‐related genes. GO terms and KEGG with *P* < 0.05 were considered significantly enriched by differential methylation loci‐related genes.

## 3 RESULTS

### 3.1 Micro‐CT scanning of ossified ligamentum flavum in single‐ and multiple‐level TOLF

**FIGURE 1:**
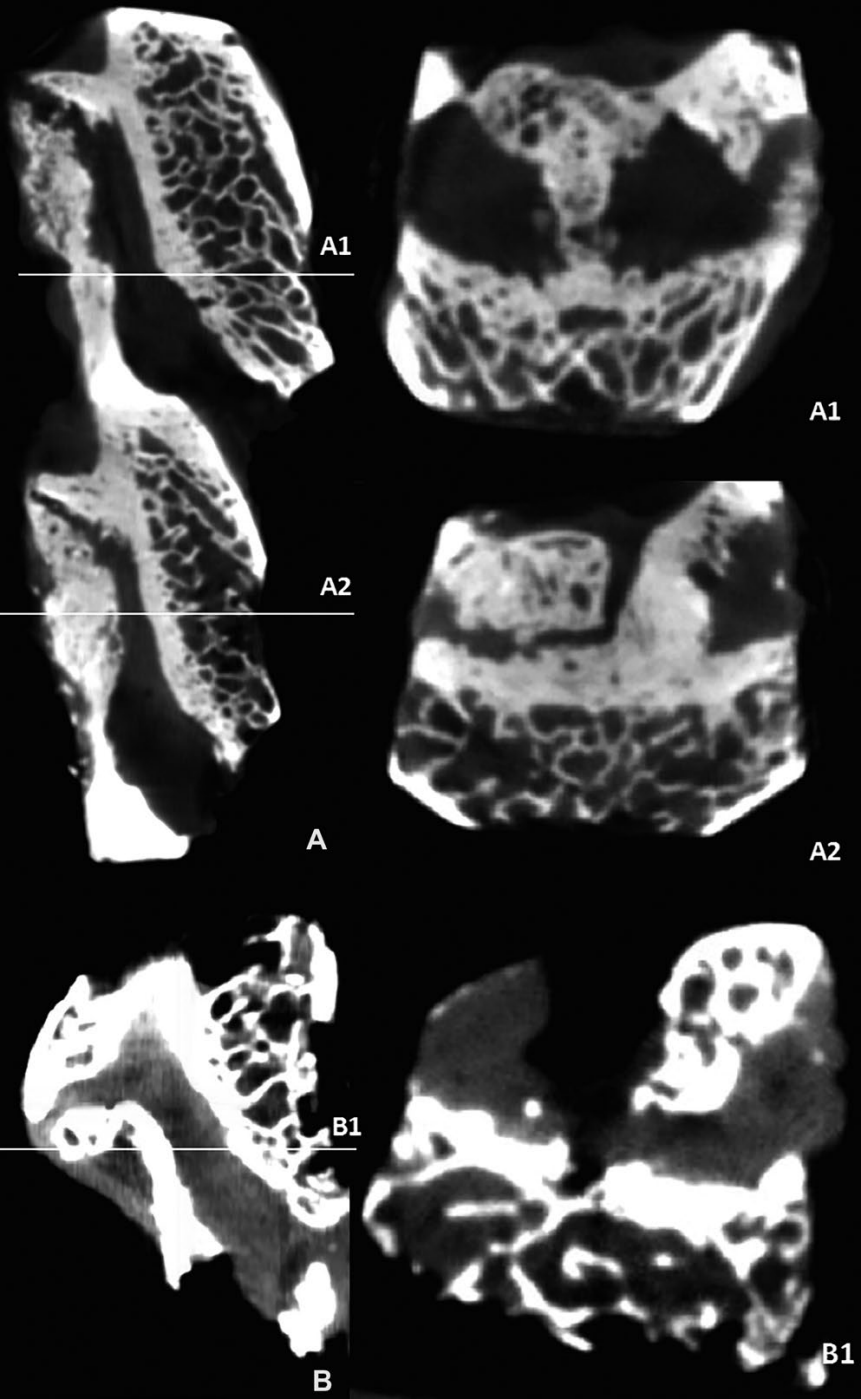
Micro‐CT images of TOLF ossification. A, Micro‐CT images of multiple‐level TOLF and its horizontal section A1and A2, (B) micro‐CT images of single‐level TOLF and its horizontal section B1. CT, computed tomography; TOLF, thoracic ossification of the ligamentum flavum

Sixteen patients were enrolled in this study, that is, eight patients each with single‐ and multiple‐level TOLF. As shown in Figure 1, the ossification of the ligaments seemed to arise at the attachments with adjacent lamina and correlate with the orientation of the zygapophyseal joints. Moreover, different ossification patterns and degrees of ligamentum flavum were observed. Immature and mature ossification^5^ of the ligamentum flavum were observed either alone or in combination at each joint of the upper and lower lamina. Unilateral and bilateral ossified ligament flavum were seen, but one side of the ossification often appeared more serious than the other.

### 3.2 Single‐ and multiple‐level TOLF groups showed distinct methylation profiles

A total of 8 466 150 CpG sites were analysed in this study. All data of the differentially methylated CpGs, unique genes, hypermethylated loci, hypomethylated loci, hypermethylated genes and hypomethylated genes are listed in Table S3. We detected 550 differentially methylated CpGs in the multiple‐healthy group, including 372 hypermethylated loci and 178 hypomethylated loci. Meanwhile, there were 572 differentially methylated CpGs, including 375 hypermethylated loci and 197 hypomethylated loci in the single‐healthy TOLF group. The details and full list of differentially methylated CpG sites were shown in the [Link], [Link], [Link].

**FIGURE 2:**
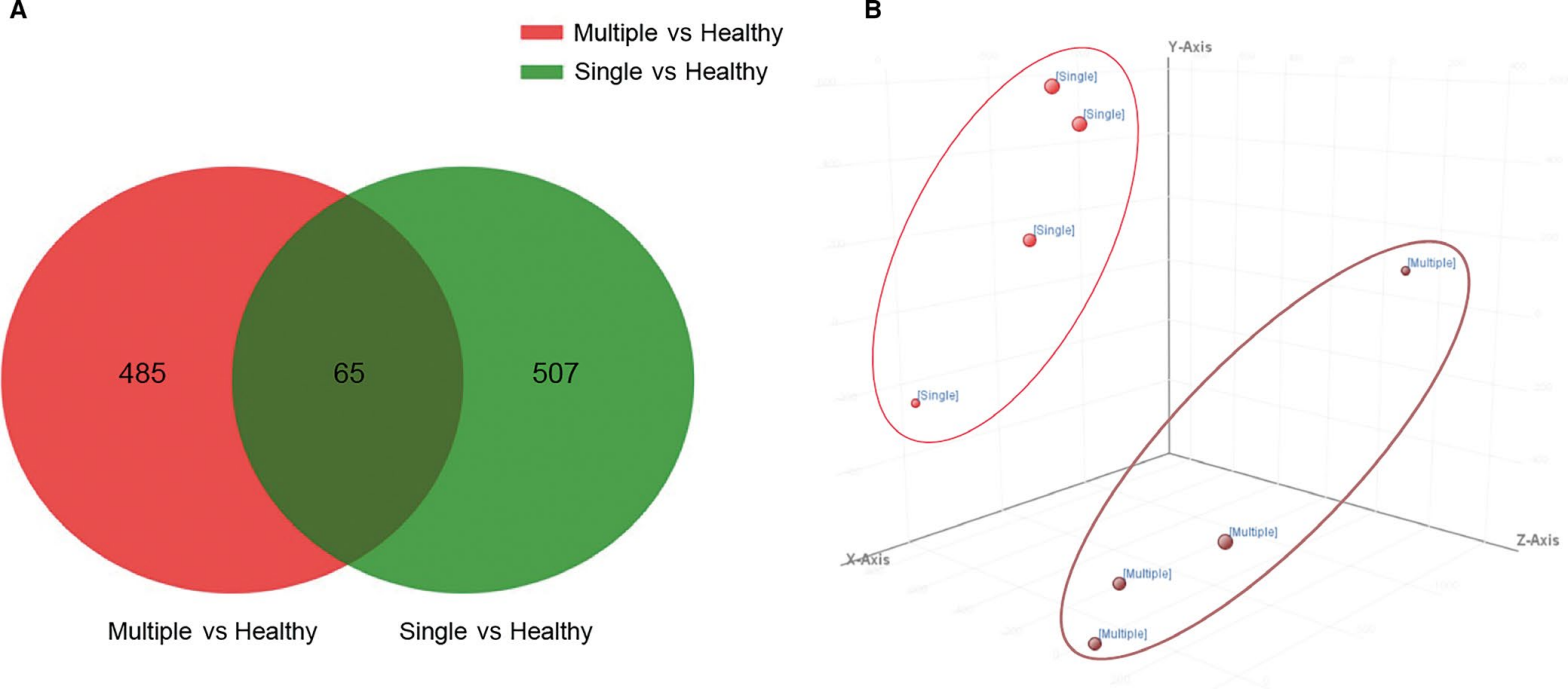
Principal component analysis (PCA) and Venn diagram showed distinct methylation profiles of single‐ and multiple‐level TOLF groups. A, Venn diagram showing the overlapping differentially methylated CpGs in subjects with multiple‐ and single‐level TOLF. B, PCA of DNA methylation data. The red dots indicate the single‐level TOLF samples, while the brown dots indicate the multiple‐level TOLF samples. PCA revealed clear clustering based on TOLF type. CpGs, cytosine‐phosphate‐guanine dinucleotides; TOLF, thoracic ossification of the ligamentum flavum

**FIGURE 3:**
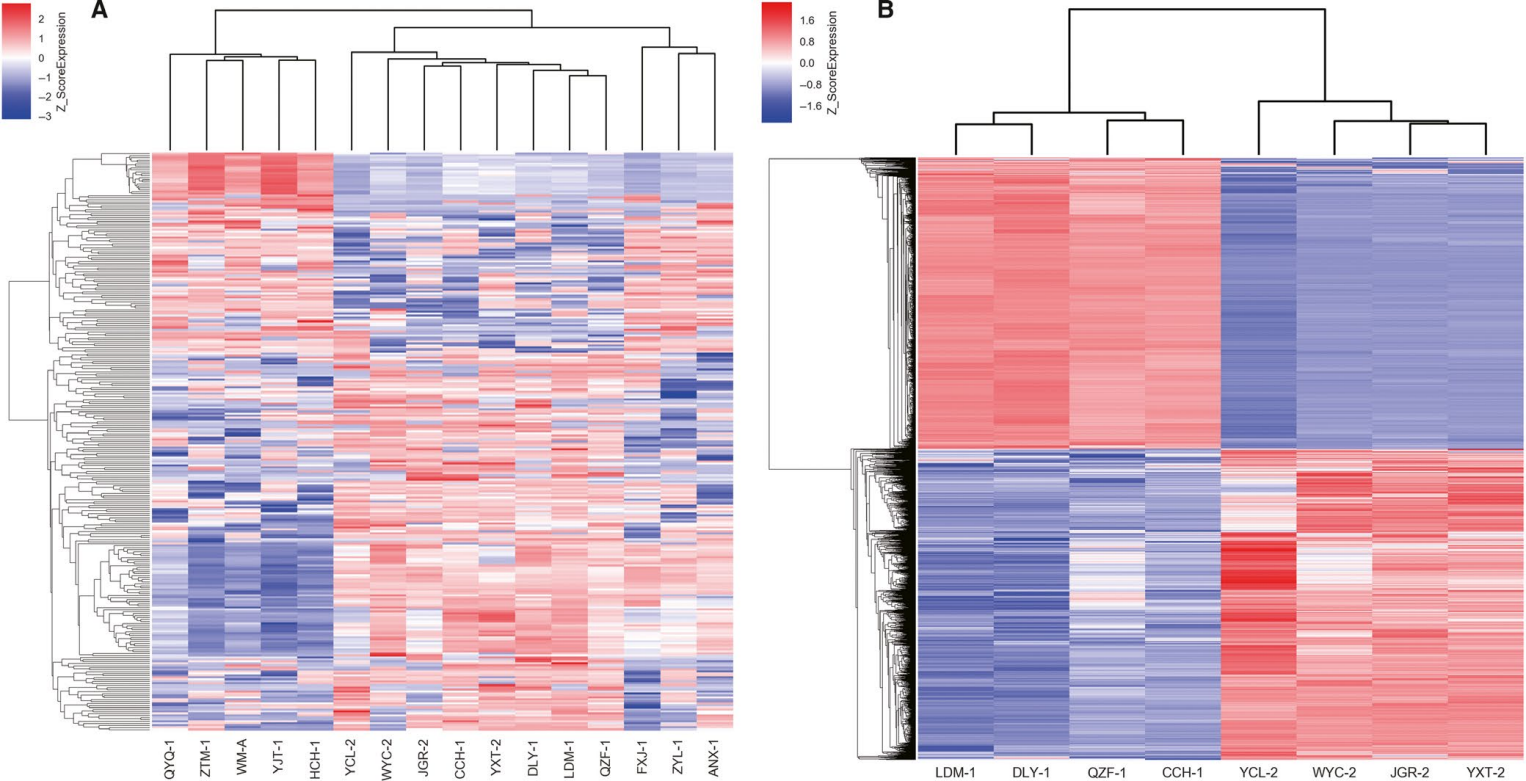
Heat map of multiple‐level TOLF group and single‐level TOLF group. A, Heat map of hierarchical clustering of 4 multiple‐, 4 single‐level patients and healthy controls by beta values of the differentially methylated CpGs, (B) heat map of hierarchical clustering of multiple‐ and single‐level TOLF by beta values of the differentially methylated CpGs. CpGs, cytosine‐phosphate‐guanine dinucleotides; TOLF, thoracic ossification of the ligamentum flavum

Next, we overlapped the differentially methylated CpGs from multiple‐healthy group and single‐healthy group, only 65 differentially methylated CGs common to these two groups were identified (Figure 2A). These data indicated distinct DNA methylation profiles in the two types of TOLF. By means of principal component analysis (PCA), we observed two distinct clusters of samples reflecting the TOLF types (Figure 2B). Then, we performed two hierarchical clustering analyses based on the beta values of the differentially methylated CGs in multiple + single/healthy group and multiple‐single group (Figure 3). The heat map and hierarchical clustering analyses showed that the TOLF patients and healthy controls have distinct epigenetic landscapes (Figure 3A). Meanwhile, the two subtypes of TOLF (multiple‐ and single‐level TOLF) also showed different features of DNA methylation profile (Figure 3B).

### 3.3 GO and pathway enrichment analysis

We further identified differentially methylated genes (hypermethylated and hypomethylated) that were significantly enriched in the two TOLF types. GO terms and KEGG with *P* < 0.05 were considered significantly enriched by differential methylation loci‐related genes. The full lists were shown in the [Link], [Link], [Link]. After reviewing the research articles about TOLF, we shortlisted the differentially methylated GO terms functionally related to bone development, osteogenesis and inflammatory processes (Table 2).

**TABLE 2:** GO enrichment analysis results of the differentially methylated genes related to osteogenesis and inflammation

Trend	Term_ID	Term_description	FoldEnrichment	*P*_value
M‐H				
Up	GO:0048704	Embryonic skeletal system morphogenesis	13.16586538	2.90E‐06
	GO:0048863	Stem cell differentiation	14.1757863	0.00032061
	GO:0031098	Stress‐activated protein kinase signalling cascade	9.444205084	0.00126798
	GO:0035994	Response to muscle stretch	14.55512972	0.00175463
	GO:0035902	Response to immobilization stress	12.25471698	0.00271042
Down	GO:0060272	Embryonic skeletal joint morphogenesis	35.62987013	0.0019721
	GO:0050896	Response to stimulus	11.78325359	0.0026735
S‐H				
Up	GO:0035023	Regulation of Rho protein signal transduction	8.08909445	0.00016791
	GO:0032966	Negative regulation of collagen biosynthetic process	29.79821429	0.00316157
	GO:0060712	Spongiotrophoblast layer development	26.071875	0.00392708
Down	GO:0060348	Bone development	9.576923077	0.00117239
	GO:0035023	Regulation of Rho protein signal transduction	6.083858998	0.00194509
	GO:0050778	Positive regulation of immune response	16.81511747	0.00837993
	GO:0000165	MAPK cascade	4.381818182	0.01581912

Abbreviations: Down, down‐methylated genes; GO, Gene Ontology; M‐H, multiple‐healthy group; S‐H, single‐healthy group; Up, up‐methylated genes; MAPK, mitogen‐activated protein kinase.

### 3.4 Pyrosequencing validation

**FIGURE 4:**
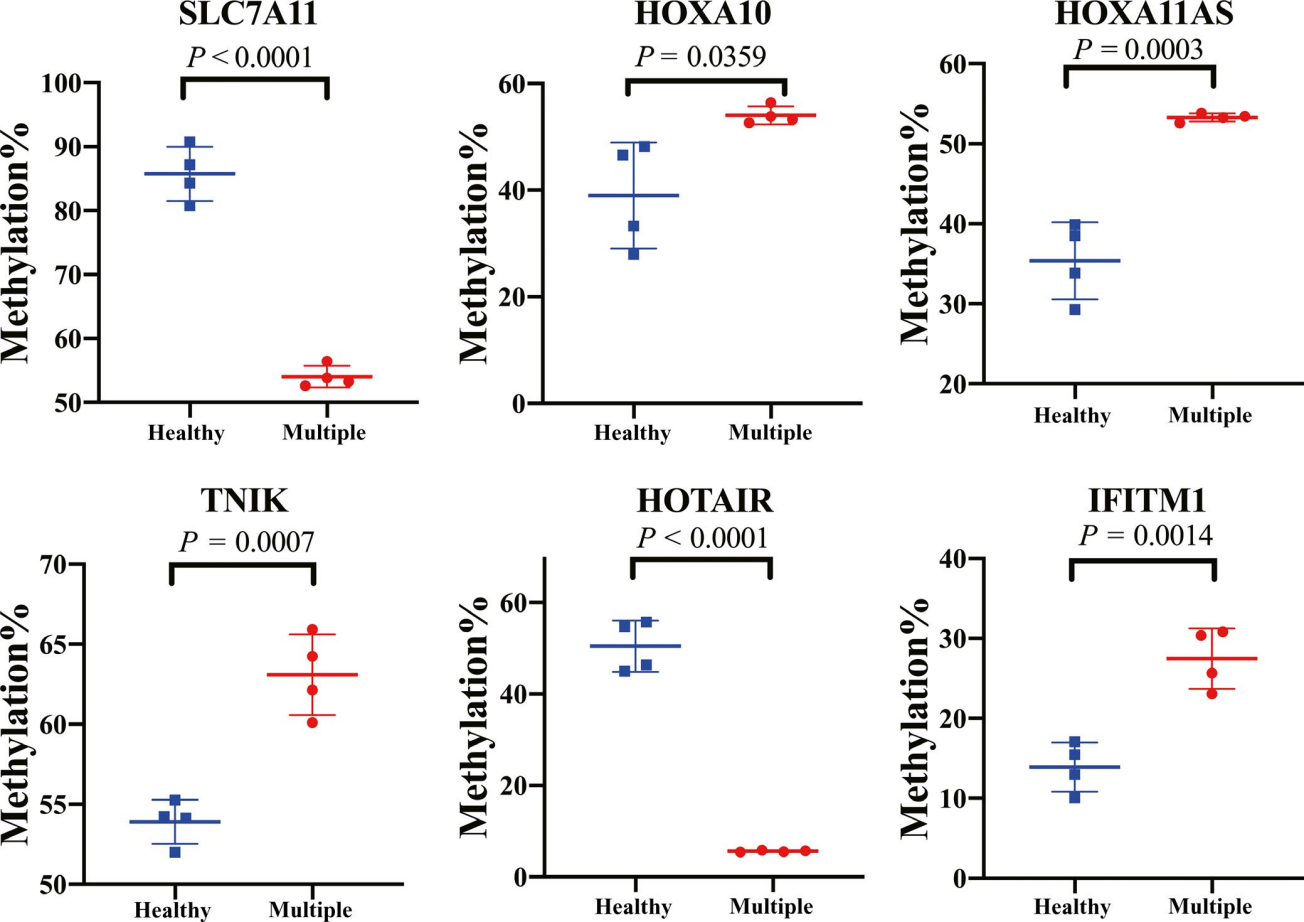
Validation of the results of the Illumina 850K DNA methylation bead array analysis by pyrosequencing. Correlations between Illumina 850K array data and pyrosequencing analysis. Representative data for CpG sites in the *SLC7A11*, *HOXA10*, *HOXA11AS*, *TNIK*, *HOTAIR* (multiple‐healthy group) and *IFITM1* (single‐healthy group) genes are shown. CpGs, cytosine‐phosphate‐guanine dinucleotides

The number of genes that were validated was limited by the remaining amount of DNA. We therefore selected several genes with a potential role in bone biology, osteogenesis and inflammation, and validated differential methylation by pyrosequencing, including *SLC7A11*, *HOXA10*, *HOXA11AS*, *TNIK*, homeobox transcript antisense RNA (*HOTAIR*) in the multiple‐healthy TOLF group and *IFITM1* in the single‐healthy TOLF group, listed in Table 3. We selected six most significantly differentially methylated CpGs from all the differentially methylated CpGs of these genes, which were then validated by pyrosequencing, and all the CpG sites showed different methylation level in their groups (Figure 4).

**TABLE 3:** Differentially methylated genes related to osteogenesis and inflammation

Gene name	Target‐ID	UCSC accession	UCSC group	Diff score	Control vs case
M/H					
*SLC7A11*	cg24676461	NM_014331	Body	−54.62678	***
*HOXA10*	cg10724867	NM_153715	Body	46.73739	***
*HOXA11AS*	cg13352750	NR_002795	Body; TSS1500	−35.13517	***
*TNIK*	cg03460350	NR_027767	Body	29.0048	**
*HOTAIR*	cg18040901	NR_003716	Body	−42.70643	***
S/H					
*IFITM1*	cg06632214	NM_003641	1stExon; 5ʹUTR	28.53919	**

Abbreviations: Down, down‐methylated genes; M‐H, multiple‐healthy group; S‐H, single‐healthy group; Up, up‐methylated genes; UCSC, https://genome‐asia.ucsc.edu/index.html.

** *P* < 0.01,

*** *P* < 0.001.

### 3.5 qRT‐PCR validation

**FIGURE 5:**
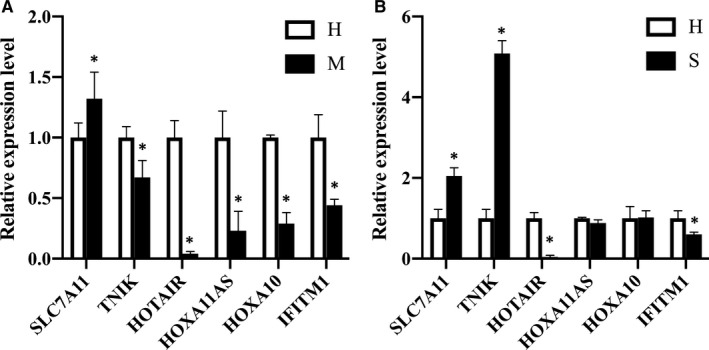
Confirmation of expression level of the selected genes by qRT‐PCR. Expression levels of selected genes in ligament flavum of multiple‐level TOLF group and single‐level TOLF group were analysed by qRT‐PCR. Gene expression levels were normalized to β‐actin. *P < 0.05. H, healthy controls; M, multiple‐level TOLF (A); qRT‐PCR, quantitative real‐time polymerase chain reaction; S, single‐level TOLF (B); TOLF, thoracic ossification of the ligamentum flavum

To further assess the functional relevance of the identified genes for TOLF, we compared the mRNA expression levels. We demonstrated significantly differential expressions of these genes from TOLF samples compared with those from non‐TOLF samples (Figure 5). This was consistent with the methylation arrays data.

## 4 DISCUSSION

Thoracic ossification of the ligamentum flavum is usually characterized by pathological heterotopic ossification in ligamentum flavum. In our previous study, we showed 24 MRI images of patients in the non‐TOLF, single‐ and multiple‐level TOLF groups with apparent distributional difference, and found the differences in osteogenic differentiation potency between single‐ and multiple‐level TOLF that may be related to the different pathogenesis and genetic background.^7^ In this study, firstly to identify the micro‐morphological characteristics of TOLF, we used micro‐CT to observe different degrees of ossification (Figure 1). The ossification patterns were discovered in single‐level and multiple‐level TOLF by micro‐CT. It was seen that the ossification of the ligamentum flavum usually arose near the attachment points of the lamina and zygapophyseal joints. The ligamentum flavum can ossify from the upper and lower sides, along the ventral side of the ligament, closed gradually, and could fuse to form a bridge‐like ossification mass. The space of the adjacent lamina was narrowed and the fusion of the ossification appeared at one side or even both sides, which may lead to joint stiffness and limited spinal activity. Two different ossification patterns were classified in previous study: immature and mature ossification,^5^ and showed that angiopoietin‐2 promotes osteogenic differentiation of thoracic ligamentum flavum cells via modulating the Notch signalling pathway. Another study showed that mechanical stress is a crucial factor for the development of TOLF,^7^ but whether uneven mechanical stress triggers the ossification of small spinal joints is still unknown.

We then observed a significant difference in the DNA methylation profiles of ligaments between patients with TOLF and healthy control subjects, suggesting the implication of methylation in the development of TOLF. The identification of the DNA methylation landscape would provide new insights into the epigenetic mechanisms in TOLF and be notably advantageous for TOLF classification, patient selection, follow‐up and early intervention. Previous report^7^ suggested that multiple‐ and single‐level TOLF have different clinical manifestations and are associated with different osteogenic differentiation potencies under conditions of cyclic mechanical stress. Therefore, we speculated that the mechanisms underlying the pathogenesis of the two types of TOLF are different, and it was necessary to compare the two types of TOLF separately. Then, based on the entire DNA methylation landscape, single‐level and multiple‐level TOLF were shown to have distinct epigenomic profiles, and the Venn diagram revealed 65 significantly differentially methylated CpGs in patients with TOLF, compared to the healthy controls. These CpGs were differentially methylated in both multiple‐ and single‐level TOLF, and then the distinct DNA methylation profiles in the two types of TOLF were confirmed by heat map construction, hierarchical clustering analysis, and PCA. These results indicated that single‐ and multiple‐level TOLF should be analysed separately in the future.

At the epigenetic level, subsequent in‐depth analysis of the CpG dinucleotides conferring these distinct profiles revealed a significant enrichment for inflammation‐related, chondrogenic, osteogenic and developmental genes on GO enrichment analysis. To further evaluate the functional relevance of the different methylated genes which relates to osteogenesis and inflammation, we selected six significantly differentially methylated CGs (five in the multiple‐level TOLF group and one in the single‐level TOLF group) and validated them by pyrosequencing (4). *SLC7A11* is involved in the osteogenic differentiation of MSCs and bone formation, and it is also known as cystine/glutamate antiporter xCT and is a predicted 12‐transmembrane protein required for amino acid selectivity.^20^, ^21^, ^22^, ^23^ *HOTAIR* has been widely validated as having an unignorable role in oncogenic progression,^24^ and it also plays a key role in regulating osteogenic differentiation and proliferation,^25^ and has been shown to be expressed in human cartilage samples.^26^ Several studies have revealed that *IFITM1* is induced by interferon‐γ during osteoblast differentiation in human bone marrow stromal stem cells^27^ and increases osteogenesis through runt‐related transcription factor 2 (RUNX2) in human alveolar‐derived bone marrow stromal cells.^28^ There were significant differences on methylation level of five CpGs in the multiple‐level TOLF group and 1 CpG in the single‐level TOLF group. These results indicated the correlation between the differential methylation level and the progress of TOLF, and showed different DNA methylation features and epigenetic mechanisms in the development of multiple‐ and single‐level TOLF.

We found that *HOX* gene clusters and its related cofactors have significantly different methylated patterns in patients with TOLF and healthy controls. Studies showed that the expression of certain sets of *HOX* genes can regulate not only the regenerative propensity of neural crest cells, but also normal skeletal development.^29^, ^30^, ^31^, ^32^ *HOXA11‐AS* is a newly identified long non‐coding RNA found in various human carcinomas and other diseases, and may regulate the inflammation induced by diabetic arteriosclerosis via the PI3K/AKT pathway.^33^ *HOXA10* controls osteoblastogenesis by activating RUNX2 and promotes bone formation via the direct regulation of osteoblast phenotypic genes.^34^ This observation suggests that *HOX* genes may participate in the ossification associated with TOLF, broadening the mechanisms whereby *HOX* genes regulate ossification. Thus, the molecular mechanism underlying the abnormal methylation levels of *HOX* genes in TOLF and the inflammatory processes involved in TOLF are yet to be studied.

In conclusion, we first conducted a comparative analysis of the genome‐wide DNA methylation profiles of patients with TOLF and non‐TOLF subjects and directly demonstrated that the DNA methylation profile was altered under conditions of TOLF. Meanwhile, according to the PCA, heat map construction and GO analysis of the genome‐wide DNA methylation profile, single‐level and multiple‐level TOLF showed significant differences, providing the potential aetiological basis for the classification of TOLF at the epigenetic level, and thus suggesting that these two subtypes of TOLF should be conducted separately in future pathogenesis studies. However, this study had a relatively small sample size, and we only confirmed the six differentially methylated CpGs among the different types of TOLF. In the future, we plan to carry out follow‐up studies to identify the function of these targeted genes and discuss the mechanism underlying the regulation of ossification by the altered epigenetic patterns. Collectively, these findings will contribute to a better understanding of TOLF.

## CONFLICT OF INTEREST

The authors declared that they have no competing interests.

## AUTHOR CONTRIBUTION

**Tianqi Fan:** Conceptualization (equal); Data curation (equal); Formal analysis (equal); Investigation (equal); Methodology (equal); Resources (equal); Software (equal); Validation (equal); Writing‐original draft (lead); Writing‐review & editing (lead). **Xiangyu Meng:** Formal analysis (equal); Funding acquisition (equal); Software (equal); Writing‐review & editing (equal). **Chuiguo Sun:** Data curation (equal); Resources (equal). **Xiaoxi Yang:** Data curation (equal); Formal analysis (equal); Software (equal). **Guanghui Chen:** Data curation (equal); Formal analysis (equal); Software (equal). **Weishi Li:** Conceptualization (equal); Investigation (lead); Methodology (lead); Project administration (lead); Resources (lead); Supervision (equal); Writing‐review & editing (equal). **Zhongqiang Chen:** Conceptualization (lead); Funding acquisition (equal); Project administration (equal); Resources (equal); Supervision (lead); Validation (equal); Writing‐original draft (lead); Writing‐review & editing (lead).

## Supporting information

Table S1‐S3

Fig S4

Fig S5

## Data Availability

Data can be obtained from the corresponding author on request.
