# Supplementary material for: Genome‐wide DNA methylation profile analysis in thoracic ossification of the ligamentum flavum
Source: J Cell Mol Med. 2020 Jun 24;24(15):8753–62. doi: 10.1111/jcmm.15509 (PMC7412700; doi:10.1111/jcmm.15509)
Supplement: Supplementary file 1 — Table S1‐S3 [file JCMM-24-8753-s001.docx]

| **Gene** | **Target ID** | Forward primer | Reverse primer | Sequence primer |
| --- | --- | --- | --- | --- |
| M-H |  |  |  |  |
| *SLC7A11* | cg24676461 | TATTTATTGATTGTTGGTTTGTTAGG | ACTAAACAATAAACCAAAATTACACAACT | GGTTTGTTAGGTTGG |
| *HOXA10* | cg10724867 | GGGTTGTGGGAGGGTAAA | CTCCAAAACTTCTACCCCTAAAA | GGGTGGTAGAAGTTTG |
| *HOXA11AS* | cg13352750 | TTGTAGTTATTTTAGGGGAAGTAATAGA | AAACCCTACAATTAAACACAAACAT | CACCCCAACCTCTCC |
| *TNIK* | cg03460350 | ATTAGTATTTTTTGGGAGATTGTTGG | AAAACATTCCACTAATCAAACTATCTCT | TTTAAATTTATTGAAGTAGAATTTG |
| *HOTAIR* | cg18040901 | ATAAAGATGGAGATGATAAGAAGAGTAAG | ATCAACCACTACCCCACA | GGGGTTTGGTGGGTT |
| S-H |  |  |  |  |
| *IFITM1* | cg06632214 | TTGGTTTTGGGGAAGGAAGT | TAATCCCTAACTAATTCACCAATTTACA | GGTGTTAGGATGTTGGGA |

Table S1 Primer sequences for pyrosequencing analysis

M-H: Multiple-Healthy Group, S-H: Single-Healthy Group, Up: up-methylated genes, Down: down-methylated genes.

Table S2 Sequences of qRT-PCR primers used for gene expression.

| Gene Symbol | Forward primer (5’-3’) | Reverse primer (5’-3’) |
| --- | --- | --- |
| SLC7A11 | TATCCCTGGCATTTGGACG | AGCTGTAATGAGCTTGATCG |
| HOXA10 | GGCTTGACTTTGGCTGATT | AGCTGGGATATCTTACAGAGG |
| HOXA11AS | GTTTGAAGCCGTGGATGT | GTGACCATGAATGAGAGAGTG |
| TNIK | GTATGTAAACACCTATGGCCG | CGGATCTCAATAGCTTTCTCG |
| HOTAIR | ATCTGATTTGGTGTTCCATGAG | CGTTCATGTGGCGAGCTA |
| IFITM1 | AACCACACTTCTCAAACCTTCA | CACAGCCACCTCATGTTC |
| ACTB (Actin Beta) | CATTCCAAATATGAGATGCGTT | TACACGAAAGCAATGCTATCAC |

M-H: Multiple-Healthy Group, S-H: Single-Healthy Group, Up: up-methylated genes, Down: down-methylated genes.

Table S3 Total data of epigenomic landscape

|  | Multiple+Single VS Healthy | Multiple VS Single | Multiple VS Healthy | Single VS Healthy |
| --- | --- | --- | --- | --- |
| differentially methylated CpGs | 289 | 4208 | 550 | 572 |
| unique genes | 136 | 2352 | 273 | 309 |
| hypermethylated loci | 189 | 2172 | 372 | 375 |
| hypomethylated loci | 100 | 2036 | 178 | 197 |
| hypermethylated genes | 85 | 985 | 178 | 104 |
| hypomethylated genes | 51 | 1470 | 98 | 214 |

M-H: Multiple-Healthy Group, S-H: Single-Healthy Group, Up: up-methylated genes,

Down: down-methylated genes.
